# Shrink-Induced Superhydrophobic and Antibacterial Surfaces in Consumer Plastics

**DOI:** 10.1371/journal.pone.0040987

**Published:** 2012-08-20

**Authors:** Lauren R. Freschauf, Jolie McLane, Himanshu Sharma, Michelle Khine

**Affiliations:** 1 Department of Biomedical Engineering, University of California Irvine, Irvine, California, United States of America; 2 Department of Chemical Engineering and Materials Science, University of California Irvine, Irvine, California, United States of America; RMIT University, Australia

## Abstract

Structurally modified superhydrophobic surfaces have become particularly desirable as stable antibacterial surfaces. Because their self-cleaning and water resistant properties prohibit bacteria growth, structurally modified superhydrophobic surfaces obviate bacterial resistance common with chemical agents, and therefore a robust and stable means to prevent bacteria growth is possible. In this study, we present a rapid fabrication method for creating such superhydrophobic surfaces in consumer hard plastic materials with resulting antibacterial effects. To replace complex fabrication materials and techniques, the initial mold is made with commodity shrink-wrap film and is compatible with large plastic roll-to-roll manufacturing and scale-up techniques. This method involves a purely structural modification free of chemical additives leading to its inherent consistency over time and successive recasting from the same molds. Finally, antibacterial properties are demonstrated in polystyrene (PS), polycarbonate (PC), and polyethylene (PE) by demonstrating the prevention of gram-negative *Escherichia coli* (*E. coli*) bacteria growth on our structured plastic surfaces.

## Introduction

The spread of bacteria is a common problem and is the main source of health associated infections. In 2009, such health associated infections cost the healthcare industry $28–45 billion and ranged from food poisoning to septicemia, often leading to extensive hospital care and even death [1], [2]. Bacterial exposure can occur during surgical procedures or can be transferred patient-to-patient from infected hospital surfaces [3]. Hospitals are a major source of bacterial spread, but everyday facilities also act as distributors of bacterial disease. Flores et al. has shown that public restrooms house at least nineteen strains of bacteria, ranging from skin, gut, and soil sources that can be transferred by touch [4]. Furthermore, multiple bacterial strands are capable of growing on plastics and fabric surfaces for days and even months [4]–[7]. Therefore, there is a growing demand for reliable antibacterial surfaces to combat this common occurrence of contamination.

Currently, there are fabrication methods for antibacterial reagents and structurally modified antibacterial surfaces. Silver nanoparticles have been used as a bacterial growth inhibitor as the heavy metals disrupt and inactivate the proteins in bacteria, preventing growth [8], [9]. Functional groups on self-assembled gold monolayers have also been used to decrease bacterial motility and attachment, preventing cell adherence, growth of bacteria on surfaces, and the formation of biofilms [10]. Zheng et al. has shown that high molecular weights of chitosan inhibit gram-positive bacteria such as *Staphylococus aureus* due to lack of nutrient adsorption whereas low molecular weights of chitosan inhibit gram-negative bacteria such as *E. coli* due to a disturbed metabolism [11]. Chemically modified superhydrophobic surfaces have also been shown to inhibit bacterial growth because of the low surface energy and minimal contact with the surface for bacterial adhesion [12]. While many antibacterial reagents and chemicals effectively inhibit the growth of bacteria, they can lead to bacterial resistance and become ineffective over time [13]. Purely structural antibacterial surfaces, however, do not induce bacterial resistance and are therefore ideal for preventing the spread of infectious bacteria. Superhydrophobic surfaces have become particularly desirable as stable antibacterial surfaces because of their self-cleaning and water resistant properties.

Such superhydrophobic surfaces in nature include the lotus leaf [14], springtails (*Collembola*, *Entognatha*) [15], and termite wings (*Nasutitermes* sp.) [16] which demonstrate properties such as self-cleaning, bacterial resistance, and flight efficiency. Superhydrophobicity can be achieved artificially through structural [17], [18] or chemical [19], [20] alterations to allow for free movement of water across a surface due to water's high contact angle (CA) and low sliding angle (SA). Current fabrication techniques employ complex production methods such as photolithography [21], [22], chemical vapor deposition [23], and self assembled monolayers [24] to create highly organized structures. It is also possible for heterogeneous micro and nanoscale structures to yield superhydrophobicity using gels, colloids, and oxides [12], [17], [18], [25]. However, all of these methods pose a time consuming and costly barrier to production. By simplifying the fabrication process and enabling its scale up and its structural integration into existing surfaces, the benefits of superhydrophobic surfaces can be readily available to a range of materials for various biomedical (e.g. implants, coatings) as well as consumer applications.

In this study, we create multi-scale structures ranging from the nano to the micro-range by leveraging the buckling of metal coated shrink film; these structures can be readily transferred into any plastic using a rapid cast and mold method, resulting in superhydrophobic surfaces in hard plastics for antibacterial applications. Hard plastics such as PS, PC, and PE are commonly used in commercial applications because they are nonreactive, are biocompatible [26], [27], and can be manufactured using inexpensive techniques such as roll-to-roll manufacturing. Polydimethylsiloxane (PDMS), a widely used polymer for sealing, coating, and molding [28], is used as a mold for casting because of its thermal stability and the ability to imprint high aspect ratio and high resolution features with good fidelity. The superhydrophobic properties are achieved without chemical alteration. With the initial substrate, we are able to produce multiple superhydrophobic PDMS casts for molding. Each of these superhydrophobic PDMS substrates is capable of imprinting roughened features into the aforementioned hard plastics, creating a substantial number of superhydrophobic hard plastics from an initial mold. The final superhydrophobic hard plastics utilize non-wetting properties to induce antibacterial effects, which could be highly beneficial for commercial application.

### Theory

The phenomenon of superhydrophobicity is explained in part by a triad of equations centered upon the contact of water with the surface. The surface tension created between water and a surface can be calculated using Young's equation [29] where the three interfaces, solid-vapor (*λ_SV_*), solid-liquid (*λ_SL_*), and liquid-vapor (*λ_LV_*), describe the material's resulting water CA (*θ_Y_*) during thermodynamic equilibrium (1).
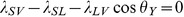
(1)In particular, as the solid-liquid surface tension increases, the CA increases due to less physical contact [30]. Further analysis of wetting can be performed with Wenzel's theory [31] where the roughness factor (*r*), determined by a ratio of the geometric surface to the apparent surface, is directly associated with the change in CA (*θ_W_*) of the roughened surface (2).
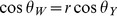
(2)In more general terms, this equation explains the ability to increase hydrophobicity on hydrophobic surfaces and increase hydrophilicity on hydrophilic surfaces merely through roughening the surface. However, another model was developed by Cassie and Baxter [32] in which water can only contact the peaks of the roughened surface versus wetting the entire surface in the Wenzel model. This occurs due to the formation of air pockets between the water and surface, decreasing the contact between the solid and liquid phases. For multi-scale (nano to micro) roughness substrates such as the lotus leaf, the Cassie-Baxter model better predicts the equilibrium state [14]. Here, the CA on the roughened surface (*θ_C_*) is additionally described by the fraction of the droplet directly in contact with the solid surface (Φ) (3).
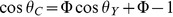
(3)The increase in solid-liquid surface tension is the primary key to creating superhydrophobicity or the lotus effect.

Superhydrophobicity is achieved when the CA exceeds 150° and the SA is reduced to less than 10°. The high surface tension, minimal surface contact, and ease of movement exhibited by water on superhydrophobic surfaces can be attributed to the presence of multiscale structures [33]. Cheng et al. demonstrated the importance of these features on the lotus leaf by removing the nanostructures which resulted in a decrease in water contact angle [14]. Furthermore, surfaces must be inherently hydrophobic [31] and have a low surface energy [34] to become superhydrophobic when structurally modified.

Thus, leveraging these superhydrophobic surfaces for antibacterial applications is feasible. Due to the minimal solid-liquid contact, the inherently low surface energy of the material, and low SA of the substrate, bacteria prefer to remain in solution rather than adhere to the surface [35], [36]. When a droplet containing bacteria contacts a superhydrophobic surface, there is minimal contact where the bacteria can adhere to the surface. Additionally, in this low contact area, there is low surface energy which allows only weak interactions between the surface and bacteria, preventing bacterial adhesion [37]. Since the superhydrophobic surface also has a low SA, bacteria in solution easily slide off the surface when tilted and do not adhere to the surface. Privett et al. even show that structural modification dominates over chemically modified hydrophobic surfaces such as fluorination for antibacterial properties [12]. With solely a structural modification, a superhydrophobic surface will repel bacteria in solution rather than kill them, negating the potential for resistance as would occur due to chemical reagents.

## Materials and Methods

### Structurally Modified Superhydrophobic Surfaces

**Figure 1:**
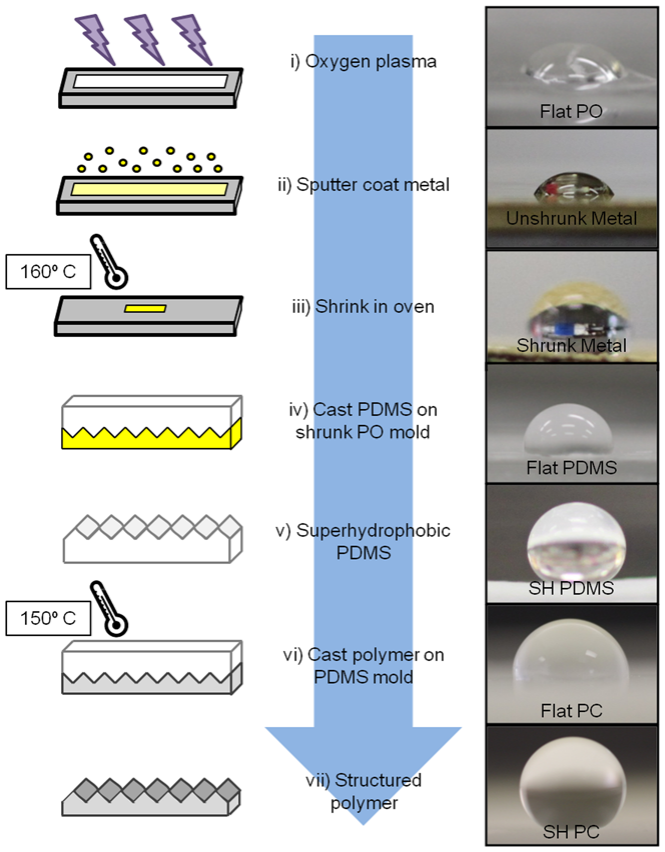
Process flow of the superhydrophobic substrates formed from shrink film paired with their respective CA. (**i**) PO film is plasma treated with oxygen for 30 seconds (**ii**) Treated PO film is sputter coated with 60 nm of silver and 60 nm of gold (**iii**) PO film is shrunk at 160°C to induce buckling and folding (**iv**) PDMS is poured over fully shrunk PO film for casting (paired photo features flat PDMS) (**v**) Superhydrophobic PDMS cast is removed from shrunk PO (**vi**) Hard plastics are casted into superhydrophobic PDMS mold by applying pressure and heat (paired photo features flat PC) (**vii**) Superhydrophobic PC casted from superhydrophobic PDMS.

By utilizing a novel shrink method, superhydrophobic hard plastics were created from shrink film, pre-stressed polyolefin (PO). PO (Sealed Air) was first pretreated with oxygen plasma (SPI Supplies) for 30 seconds to temporarily increase the surface energy for better adhesion and was then sputter coated (Quorom) with 60 nm of silver and 60 nm of gold (Figure S1). After the bimetallic coating, the PO film was heated to 160°C, causing the PO to fully shrink. While the PO shrinks due to heating, the metallic films at the surface buckle and fold, creating extremely rough, high-aspect, and multiscale structures [38]. PDMS (Dow Corning Co.) is used to cast these features into a thermally and mechanically stable medium. These features are further transferred into the hard plastics PS (Grafix Plastics), PC (McMaster-Carr), and PE (McMaster-Carr). To produce structurally modified PS, pre-stressed PS was heated to 135°C to fully shrink the polymer and then casted to the superhydrophobic PDMS mold by applying uniform pressure and heat at 150°C [39]. The PC and PE were produced using the same casting technique at 150°C. Figure 1 depicts a brief process flow of this fabrication method paired with CA images for each step.

The superhydrophobic properties of the structurally modified substrates and the original flat substrates were characterized with CA and SA measurements. A contact angle meter (Drop Shape Analysis System DSA100, KRUSS) was used to measure the CA of initial PDMS molds. Further CA measurements were taken with a drop analysis program [40] on PS, PC, and PE. The SA measurements were performed using a tool clamp with a 90° rotational arm.

### Antibacterial Surfaces

Antibacterial testing was performed on equally sized PS, PC, and PE samples for both flat and superhydrophobic substrates using DH5-α gram-negative *E. coli*. *E. coli* was inoculated in 10 mL of Luria Broth (LB) (Difco) overnight in an air bath shaker (Environ Shaker) at 37°C and 300 rpm to reach the exponential growth phase. The bacteria was then diluted 1,000× or 10,000× in LB. Using the spread plate method, plating concentrations were determined as 10^5^ colony forming units (CFU)/mL for PS and PC and 2.6×10^4^ CFU/mL for PE. For testing antibacterial properties, 10 µL of bacterial solution was placed on the surface of each substrate. Substrates were tilted at 90° to allow bacterial solution to roll off, if possible. Subsequently, samples were either rinsed with 50 µL of sterile phosphate buffered saline (PBS) or not rinsed. The surfaces of the substrates were then stamped face-down in agar (Fisher Scientific) plates to transfer residual bacteria. 50 µL of PBS was added to the agar dish to aid in spreading, and bacteria was spread using a sterile glass loop and a turntable per the spread plate method. 10 µL of bacterial solution was added directly to the control agar plates along with 50 µL of sterile PBS for performing the spread plate method. The agar plates were incubated for 24 hours at 37°C in a humidified incubator (VWR Scientific Products). Images were taken after 24 hours, and CFU counts were performed to compare bacterial growth. Agar was prepared prior to experiments according to the manufacture's protocol.

## Results

### Structurally Modified Superhydrophobic Surfaces

**Figure 2:**
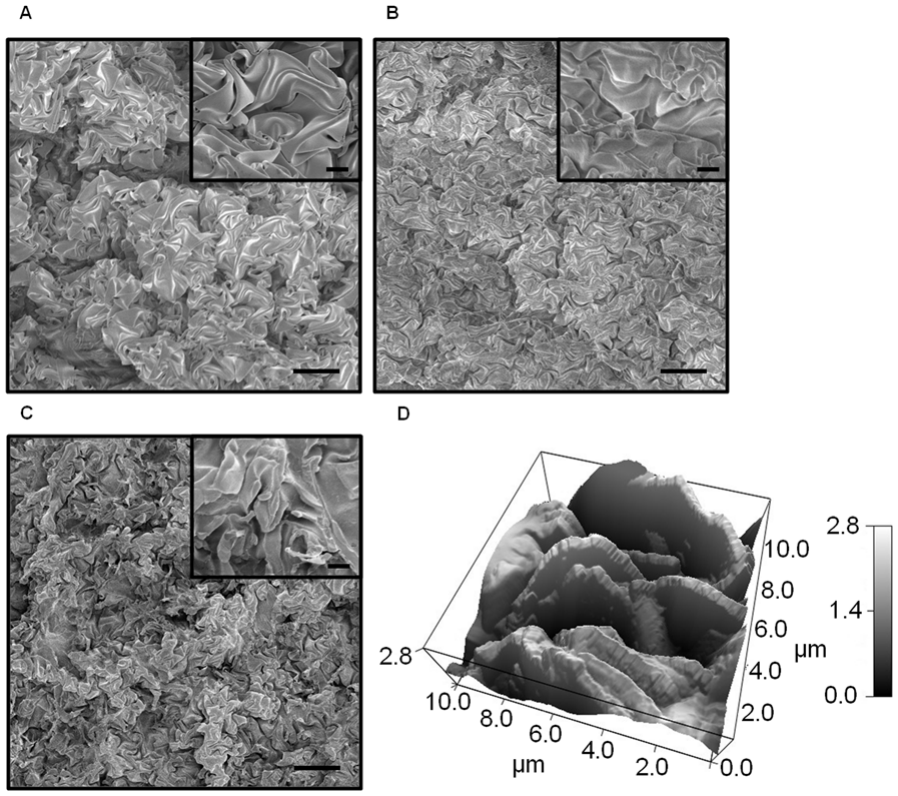
Top down SEM images and AFM of the structurally modified surfaces' multiscale structures were taken. Features are shown in (**A**) shrunk, bimetallic PO, (**B**) transferred in PDMS, and (**C**) imprinted in PS from PDMS. Scale bar is 10 µm for the large SEM images and 2 µm for the insets. (**D**) AFM 3D image of the morphology and height profile.

The heterogeneous nano and microstructures of the metal, PDMS, and PS were analyzed using a scanning electron microscope (SEM) (Hitachi S-4700-2 FES) shown in Figure 2(A–C). The roughness from the shrunk, bimetallic PO mold is translated directly into the PDMS and subsequently into the PS, PC, and PE. Nanostructures can be seen on the surface of the microstructures, leading to the enhanced hydrophobicity explained by the Cassie-Baxter theory [32]. Further visualization of morphology and height was achieved using Atomic Force Microscopy (AFM) (Asylum MPF3D), shown in Figure 2(D), displaying a three dimensional view of the shrunk, bimetallic PO mold with a heterogeneous microstructure height range of 2.8 µm and a root mean square (RMS) value of 700 nm.

**Figure 3:**
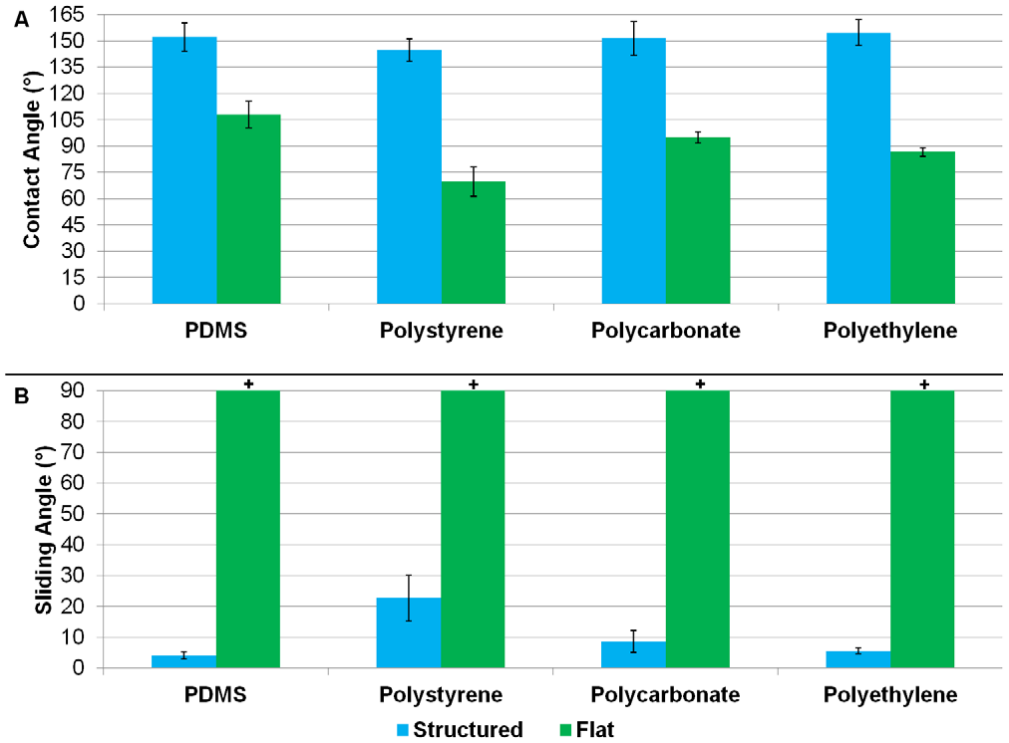
Graphs depicting CA and SA for the structurally modified surfaces compared to flat. (**A**) Contact angle measurements of structurally modified and flat PDMS, PS, PC, and PE. (**B**) Sliding angle measurements of structurally modified and flat PDMS, PS, PC, and PE. **+**represents measurements >90°.

**Figure 4:**
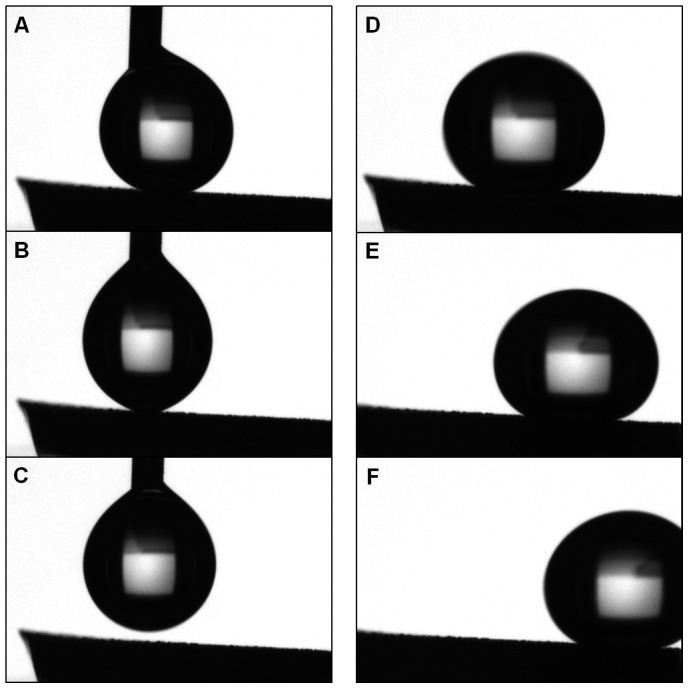
The low SA of superhydrophobic PDMS allows the water droplet to easily roll off the surface. (**A–C**) A droplet being placed on the surface of superhydrophobic PDMS retracts onto the dropper. (**D–F**) A droplet rolling off the same surface immediately after placement at a 5° angle.

CAs averaged above 150° with a maximum of 167° measured with the KRUSS system, and the average SA was below 5° with a minimum of less than 2° in PDMS, as shown in Figure 3. PC and PE yielded similarly high CAs and low SAs indicative of superhydrophobicity. PS produced slightly lower CAs and higher SAs but showed hydrophobic enhancement from its flat comparison. The low SA of superhydrophobic PDMS is depicted in Figure 4 and Video S1.

Over the course of three casts from the shrunk, bimetallic PO to PDMS, the CA remained consistently above 150° (data not shown). In addition, casting PS, PC, or PE from a single PDMS mold has yielded superhydrophobic substrates for more than 30 casts. The thermal stability of the superhydrophobicity in PDMS molds was also investigated and remained stable across a range of heat exposure from 25–100°C. PDMS samples were placed on a hotplate at 10°C intervals and allowed to acclimatize to the indicated temperature over the course of 5 minutes with a 5 µL water droplet until CA was taken (data not shown).

Calculation of the solid fraction (Φ) from the Cassie-Baxter equation (3) can be calculated using the average flat CA (*θ_Y_*) and the average structurally modified CA (*θ_C_*) for each surface (4).
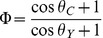
(4)The solid fraction Φ is a ratio of the properties of the structured surface to the flat surface. Since all structures are imprinted from the same initial metal PO mold to the polymers, each polymer would theoretically have the same solid fraction Φ. However, the initial *θ_Y_* is different for each polymer due to intrinsic chemical differences, causing variation in Φ between materials. Table 1 shows calculated values of Φ for our roughened substrates. The low values are similar to the findings of Zhu et al. whose calculated Φ was typically less than 0.1, indicating a highly structured surface [41]. As apparent from equations 3 and 4, as Φ approaches 0, *θ_C_* approaches 180°.

**Table 1:** Calculated values of the solid fraction (Φ) were found using the average flat CA (*θ_Y_*) and the average structurally modified CA (*θ_C_*).

Material	*θ_C_* (°)	*θ_Y_* (°)	Φ
PDMS	152	108	.17
PS	145	70	.14
PC	151	95	.14
PE	155	87	.09

A low value of Φ represents minimal water contact with the surface.

### Antibacterial Surfaces

**Figure 5:**
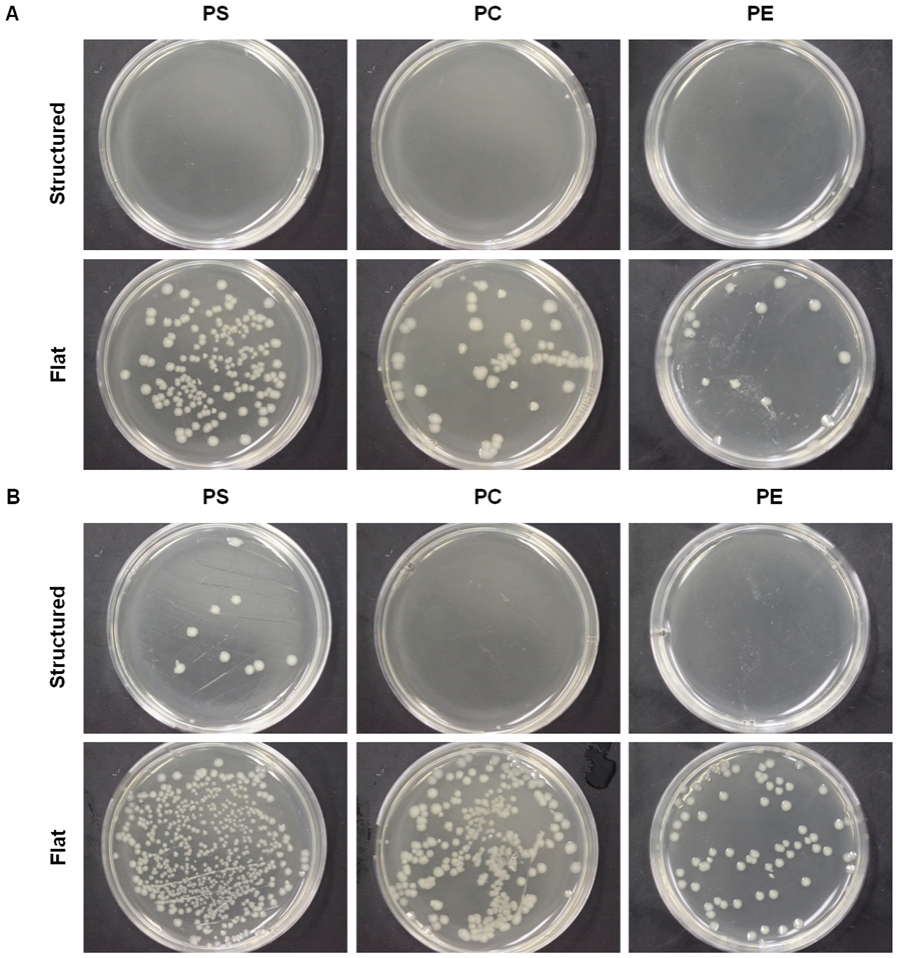
PS, PC, and PE structured and flat substrates were contaminated with a bacteria solution and either rinsed or not rinsed. The resulting bacterial growth can be observed in each plate in the form of colonies following 24 hour incubation. (**A**) Substrates were rinsed with 50 µL of PBS after bacteria solution was deposited on the surface. (**B**) Substrates were not rinsed.

Superhydrophobic surfaces exhibit a significantly reduced amount of bacterial growth over flat surfaces, as shown in Figure 5. Control agar plates of PS and PC had 100,100 CFUs, and PE had 25,800 CFUs for all conditions. Rinsed superhydrophobic surfaces yielded <100 CFUs for PS and PE, and no bacteria was observed on rinsed superhydrophobic PC (Table 2). A small fraction (<.1%) of bacteria was retained from the initial droplet on all rinsed superhydrophobic samples. The flat rinsed surfaces had much higher CFU counts where 10% of the initial number of cells placed on the flat surfaces was transferred to the agar plates even after rinsing. The no rinse superhydrophobic surfaces were also effective at preventing bacterial adhesion with only ∼2% of the original number of cells plated in the final CFU count. Not rinsed flat surfaces had ∼34% of the original number of bacteria plated. Note that all samples experienced a loss of bacteria due to gravity during the tilting step of the experiment.

**Table 2:** CFU counts for structured versus flat surfaces.

Condition	Substrate	PS	PC	PE	Adherence (Average of Experimental/Control)
Rinse	Structured	70	0	30	<0.1%
Rinse	Flat	15,700	10,700	900	10%
No Rinse	Structured	2,100	1,500	300	2%
No Rinse	Flat	>36,900*	30,700	8,900	>34%
Control	Control	100,100	100,100	25,800	100%

* One agar plate yielded a condensed area of cell growth, hindering the ability to count individual colonies. Thus, this value is an underestimate.

## Discussion

With our cast and mold method, we induced superhydrophobic properties on PDMS, PS, PC, and PE. The bimetallic layer deposited on the preshrunk PO mold provided the initial necessary mismatch in stiffness during the shrinking process to create highly structured features after complete shrinking. When casted with PDMS, the bimetallic PO mold transfers its physical shape, producing multiscale roughening on the PDMS surface and enhancing its natural hydrophobic properties. PDMS was used to imprint these features in PS, PC, and PE to yield similar heterogeneous rough structures and superhydrophobic properties.

The consistency of superhydrophobic properties is due in part to the natural properties of PDMS, PS, PC, and PE as well as the features transferred from the metal coated, shrunk PO into hard plastics. With our cast and mold method, the surface of the polymer becomes superhydrophobic due to the highly intercut high aspect ratio structures passed on from mold to cast. PDMS serves as the ideal medium to transfer these structures into the hard plastics because of its pliability yet high thermal stability. However, we found that higher levels of hydrophobicity were achieved through structural modification of initially more hydrophobic polymers (PC and PE) versus initially less hydrophobic polymers (PS). While roughening of the PS surface did increase hydrophobicity, it did not achieve characteristic values to be truly superhydrophobic because of its naturally less hydrophobic state when flat. Nevertheless, antibacterial testing for the structurally modified PS was favorable over the flat PS in both the rinse and no rinse conditions but to a lesser degree than PC and PE. Thus, for optimal hydrophobic and antibacterial surfaces, beginning with a more hydrophobic polymer seems favorable.

Superhydrophobic surfaces are antibacterial because of their minimal solid-liquid contact at the surface, weak surface interactions with bacteria, and low SA. As a result of these properties, it is energetically favorable for the bacteria to remain in solution and to roll off the surface when tilted rather than adhere to the superhydrophobic surface. This self-cleaning principle is the key to antibacterial properties of superhydrophobic surfaces. Dirt and bacteria adhere to water better than the surface and are, therefore, cleansed easily by simple rinsing, mitigating the need for antibacterial reagents. Since this antibacterial design is purely structural, a product with permanent features can be manufactured for everyday use with minimal maintenance for the customer.

This fabrication method has the potential for further development at a larger manufacturing scale and into additional materials. The PO polymer used to create the initial mold, in addition to the resulting molded hard plastics, are compatible with roll-to-roll manufacturing methods. While we demonstrate the ability to create superhydrophobic characteristics by transferring these features into only three hard plastics, this method is applicable to virtually any inherently hydrophobic plastic.

## Conclusion

Here we have presented a novel method of producing a superhydrophobic surface from PO by simply molding our unique multi-scale features into PDMS and again into the hard plastics PS, PC, and PE. This process is rapid, reproducible, and yields antibacterial surfaces on these hard plastics. By eliminating the need for chemical alterations to the surface, these superhydrophobic surfaces become much more robust due to the reliance solely on physical geometry at the surface. In addition, using PDMS as a means to transfer the superhydrophobic nano and microscale structures presents the opportunity to produce a substantial number of superhydrophobic hard plastics from a single mold. Finally, this technique is compatible with roll-to-roll manufacturing and scale-up production methods due to the use of the polymers PO, PS, PC, and PE, making this process potentially accessible for many different applications.

## Supporting Information

Figure S1 **Various thicknesses of metal deposition produce different contact angles.** To yield consistent superhydrophobicity, 60 nm of silver and 60 nm of gold was chosen as the optimal metal thickness on the PO. CAs were taken on casted PDMS.
(TIF)

Video S1 **Water does not wet the superhydrophobic surface.** Water favors the dropper rather than the superhydrophobic surface. Next, the low sliding angle of the superhydrophobic surface allows the water droplet to roll off the surface with ease. This video can also be viewed at http://shrink.eng.uci.edu/research.html.
(WMV)
