# Supplementary figures and images for: Shrink-Induced Superhydrophobic and Antibacterial Surfaces in Consumer Plastics
Source: PLoS One. 2012 Aug 20;7(8):e40987. doi: 10.1371/journal.pone.0040987 (PMC3423404; doi:10.1371/journal.pone.0040987)

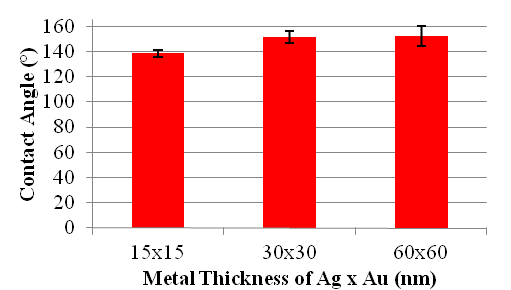

Supplement: Figure S1 — Various thicknesses of metal deposition produce different contact angles. To yield consistent superhydrophobicity, 60 nm of silver and 60 nm of gold was chosen as the optimal metal thickness on the PO. CAs were taken on casted PDMS. (TIF) [file pone.0040987.s001.tif]
